# L-selectin-dependent and -independent homing of naïve lymphocytes through the lung draining lymph node support T cell response to pulmonary *Mycobacterium tuberculosis* infection

**DOI:** 10.1371/journal.ppat.1011460

**Published:** 2023-07-05

**Authors:** Lina Daniel, Claudio Counoupas, Nayan D. Bhattacharyya, James A. Triccas, Warwick J. Britton, Carl G. Feng

**Affiliations:** 1 Immunology and Host Defence Group, School of Medical Sciences, Faculty of Medicine and Health, The University of Sydney, Sydney, Australia; 2 Centenary Institute, The University of Sydney, Sydney, Australia; 3 Charles Perkins Centre, The University of Sydney, Sydney, Australia; 4 Microbial Pathogenesis and Immunity Group, School of Medical Sciences, Faculty of Medicine and Health, The University of Sydney, Sydney, Australia; 5 The University of Sydney Institute for Infectious Diseases, The University of Sydney, Sydney, Australia; 6 Department of Clinical Immunology, Royal Prince Alfred Hospital, Camperdown, Sydney, Australia; University of Massachusetts Medical School, UNITED STATES

## Abstract

Recruiting large numbers of naïve lymphocytes to lymph nodes is critical for mounting an effective adaptive immune response. While most naïve lymphocytes utilize homing molecule L-selectin to enter lymph nodes, some circulating cells can traffic to the lung-draining mediastinal lymph node (mLN) through lymphatics via the intermediate organ, lung. However, whether this alternative trafficking mechanism operates in infection and contributes to T cell priming are unknown. We report that in pulmonary *Mycobacterium tuberculosis*-infected mice, homing of circulating lymphocytes to the mLN is significantly less efficient than to non-draining lymph node. CD62L blockade only partially reduced the homing of naïve T lymphocytes, consistent with L-selectin-independent routing of naïve lymphocytes to the site. We further demonstrated that lymphatic vessels in infected mLN expanded significantly and inhibiting lymphangiogenesis with a vascular endothelial growth factor receptor 3 kinase inhibitor reduced the recruitment of intravenously injected naïve lymphocytes to the mLN. Finally, mycobacterium-specific T cells entering via the L-selectin-independent route were readily activated in the mLN. Our study suggests that both L-selectin-dependent and -independent pathways contribute to naïve lymphocyte entry into mLN during *M*. *tuberculosis* infection and the latter pathway may represent an important mechanism for orchestrating host defence in the lungs.

## Author summary

The generation of a robust immune response relies on the efficient recirculation of naïve lymphocytes through secondary lymphoid organs, such as lymph nodes (LNs). L-selectin mediated entry of naïve lymphocytes through high endothelial venules (HEVs) has long been believed to be the main gateway for naïve lymphocytes trafficking from the blood to LNs. Here we report that this process is impaired in lung draining mediastinal LN (mLN) during Mycobacterium tuberculosis infection, which was associated with fewer HEV and disrupted expression pattern of CCL21. However, expansion of lymphatic vessels contributed to the delivery of naïve CD4^+^ T cells in an L-selectin independent manner to the mLN. Together, this study reveals that recruitment via L-selectin independent pathway is an important T cell entry route in an environment where lymphocyte trafficking via the conventional L-selectin dependent mechanism is impaired coupled with increased demand for the generation of antigen-specific effector T cells during infection.

## Introduction

Lymph nodes (LN) are specialized secondary lymphoid organs, which act as the main sites for the generation of adaptive immunity. As naïve antigen-specific T cells are present at an extremely low frequency, the initiation of an efficient adaptive immune response relies on the recruitment of a large pool of circulating naïve lymphocytes into LN to increase the probability of encounter between antigen-specific lymphocytes and their cognate antigen presented by antigen presenting cells (APCs) [1–3]. Thus, in conjunction with priming adaptive immune response, effective recirculation of naïve lymphocyte through LN underlies the critical function for these secondary lymphoid organs in immunosurveillance.

LN have two main portals of entry, high endothelial venules (HEV) and afferent lymphatic vessels ^4^. Naïve and memory lymphocytes are recruited to the LN from circulation through HEV. This recruitment process occurs via a multistep adhesion cascade that begins with the binding of L-selectin (CD62L) expressed by naïve and memory lymphocytes to peripheral node addressin (PNAd) expressed by HEV [4,5]. This mechanism is further strengthened by the interaction of CCR7 expressed on naïve lymphocytes with the arresting and homing chemokine CCL21 expressed by fibroblastic cells that line the venules [6–8]. It is generally believed that HEV act as portals that determine the entry of circulating L-selectin positive naïve lymphocytes, whereas lymphatic vessels (LV) and more specifically the afferent LV is the main pathway by which antigen, effector lymphocytes and APCs reach LN from the draining peripheral tissues [9,10]. One exception, however, has been reported for lung-draining LN where naïve T cells have been suggested to enter the LN directly via circulation and indirectly through lung tissue in naïve mice [11]. The significance of this dual-supply mechanism for the tissue-draining LN in the generation of adaptive immunity in a disease setting has yet to be investigated.

Under the homeostatic state, lymphocyte populations within the LN are maintained at a steady level. Following infection or immunization, the LN draining a site of inflammation undergoes several morphological changes that promote the increased recruitment of naïve lymphocytes [12,13]. These changes include the expansion of HEV and the arteriole that feeds into lymph node, and increased expression of CCL21 on HEV [12,14–16]. This remodelling process is thought to enhance the efficiency of recruitment and screening for rare antigen (Ag)-specific lymphocytes [12]. Defects in these processes observed in tumour models and acute viral infection lead to transiently impaired delivery of naïve lymphocytes to the LN, which in turn hinders effective immunosurveillance and immune response activation [17,18]. The impact of chronic inflammation on the patterns of naïve lymphocyte recirculation remains incompletely understood.

*Mycobacterium tuberculosis* (M.tb) is a pathogenic bacteria that establishes a persistent pulmonary infection, with the lung-draining mediastinal LN (mLN) serving as the main site for the initiation of adaptive immunity [19–21]. In this study, we report that the recruitment of naïve lymphocytes via the L-selectin-dependent HEV pathway into the mLN of M.tb infected mice was significantly less efficient than that to LN draining a non-infection site. Consequently, a substantial fraction of naïve lymphocytes entered the mLN via a L-selectin independent pathway involving afferent lymphatics. Importantly, naïve Ag-specific T cells that entered the mLN via the L-selectin-independent mechanism were responsive to cognate Ag stimulation *in vivo* and were functionally comparable to those that entered via circulation. This study reveals an L-selectin-independent CD4^+^ T cell priming mechanism in mice with chronic pulmonary infection and suggests that the operation of this additional homing pathway is critical in maintaining immune surveillance and ongoing immune response to pulmonary infection.

## Results

### The entry of circulating naïve lymphocytes to lung-draining mediastinal lymph node is reduced compared to non-draining LN in M.tb-infected mice

**Fig 1:**
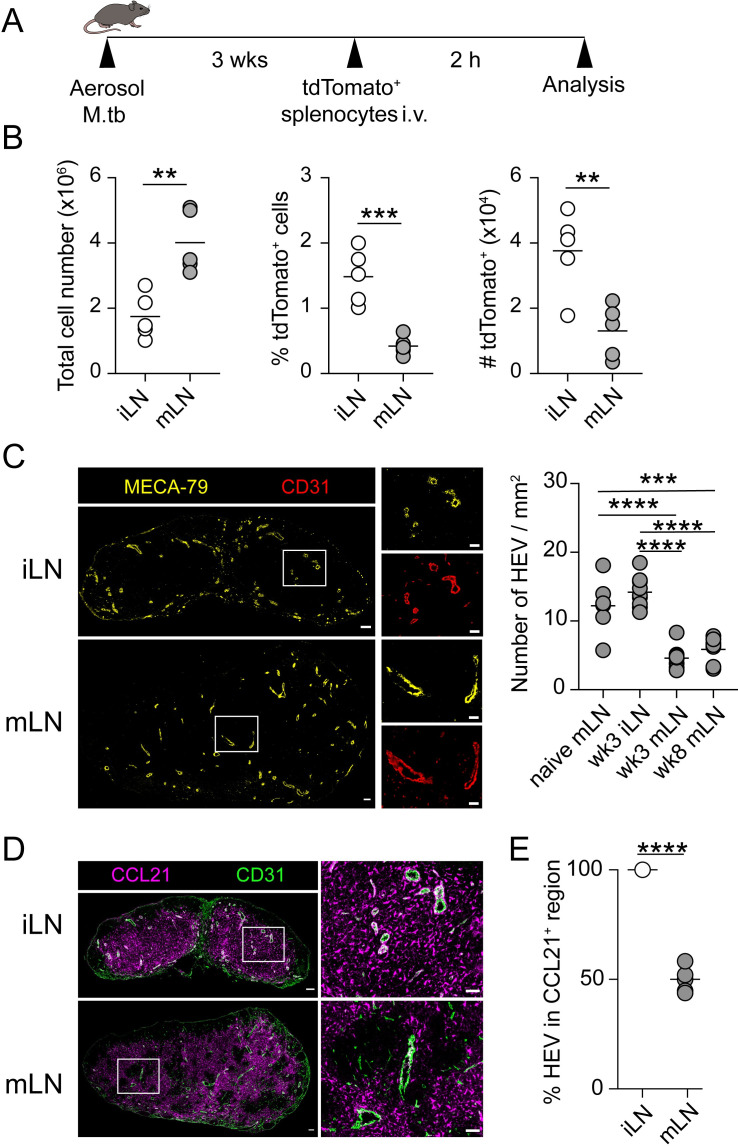
Homing of lymphocytes is impaired in the mLN during M.tb infection. (A) Experimental design. Splenocytes from tdTomato-expressing mice were injected i.v. into 3wk M.tb-infected WT mice. The iLN and mLN were analyzed 2 h post-transfer. (**B**) Total cell number of iLN and mLN as well as percentage and number of donor tdTtomato^+^ lymphocytes (2 h post cell transfer) in iLN and mLN at wk 3 p.i. (**C**) Representative staining of MECA-79 (yellow) in iLN and mLN at wk3 p.i. Scale bar 100 μm. Enlarged view of the boxed area in the left images are shown in right panels. Enlarged view representing stained HEV with MECA-79 (yellow) and CD31 (red) (Scale bar, 50 μm). Number of HEV/mm^2^ measured in naïve mLN as well as infected iLN and mLN. (**D**) Representative staining of CCL21 (magenta) and CD31 (green) in iLN and mLN at wk 3 p.i. (**E**) Percentage of HEV in CCL21 region in iLN and mLN at wk 3 p.i. Each symbol represents an individual mouse in (**B**) and sections in (**C, D**). Data in (**B**) are pooled from two independent experiments. Data in (**C**) are from two independent experiments (n = 4–6 mice / group, 1–2 sections / mouse were analyzed). Data in (**D-E**) are from two independent experiments (n = 3 mice / group, 1–2 sections / mouse were analyzed) Statistical differences between groups in (**B, E**) were determined using Student’s *t* test or in (**C**) using one-way ANOVA with Tukey’s multiple comparison test. ** *p* < 0.01, ***p < 0.001, **** *p* < 0.0001.

An important function of the lymph node is to increase the probability of encounter between rare antigen-specific T cells and their cognate antigen expressed on APCs by enabling large numbers of naïve lymphocytes circulating through the site [4]. To investigate the recirculation patterns of naïve lymphocytes in mLN during M.tb infection, we firstly examined the homing of lymphocytes into mLN versus non-draining inguinal LN (iLN). Labelled splenocytes were injected i.v. into M.tb-infected mice and their relative abundance in mLN or iLN were analyzed 2 h after cell transfer (Fig 1A). This approach has been developed to analyse lymphocyte entry in LN as the time frame is sufficient for lymphocytes to migrate from circulation to the LN, but insufficient for them to egress [22,23]. We found that despite increased cellularity of mLN compared to iLN (Fig 1B) in pulmonary M.tb-infected mice, there was a decrease in the frequency and absolute number of labelled lymphocytes that were recruited to the mLN (Fig 1B). This contrasts with some previous studies where lymphocyte recruitment via HEV was shown to increase following inflammation, with the rate of lymphocyte entry being proportional to lymph node cellularity [12,13,24].

The impaired recruitment of lymphocytes to the mLN suggested that there might be local alteration in the LN environment. Therefore, we examined the main molecules involved in lymphocytes homing expressed on HEV including, L-selectin ligand PNAd identified by MECA-79 marker, as well as the homing chemokine CCL21 (Fig 1C–1E). First, we quantified the number of HEV per LN area across entire sections of naïve mLN and mLN and iLN from M.tb infected mice at weeks 3 and 8 p.i (Fig 1C). Unexpectedly, while the density of HEV has been previously shown to increase as the LN enlarges following an infection or inflammation [14,25,26], the density of HEV in M.tb infected mLN were decreased compared to both naïve mLN and iLN. Examining CCL21 expression in iLN HEV, revealed a uniform distribution within the paracortex (Fig 1D). In contrast in mLN, CCL21 expression was variable with partial and/or complete loss of CCL21 surrounding 50% of HEV (Fig 1D and 1E). These data suggests that the entry of lymphocytes into the mLN is impaired in M.tb-infected mice, and this is associated with reduced HEV density and perturbed CCL21 expression pattern within the mLN.

### Significant proportion of naïve lymphocytes enter the mLN of M.tb-infected mice via an L-selectin-independent mechanism

**Fig 2:**
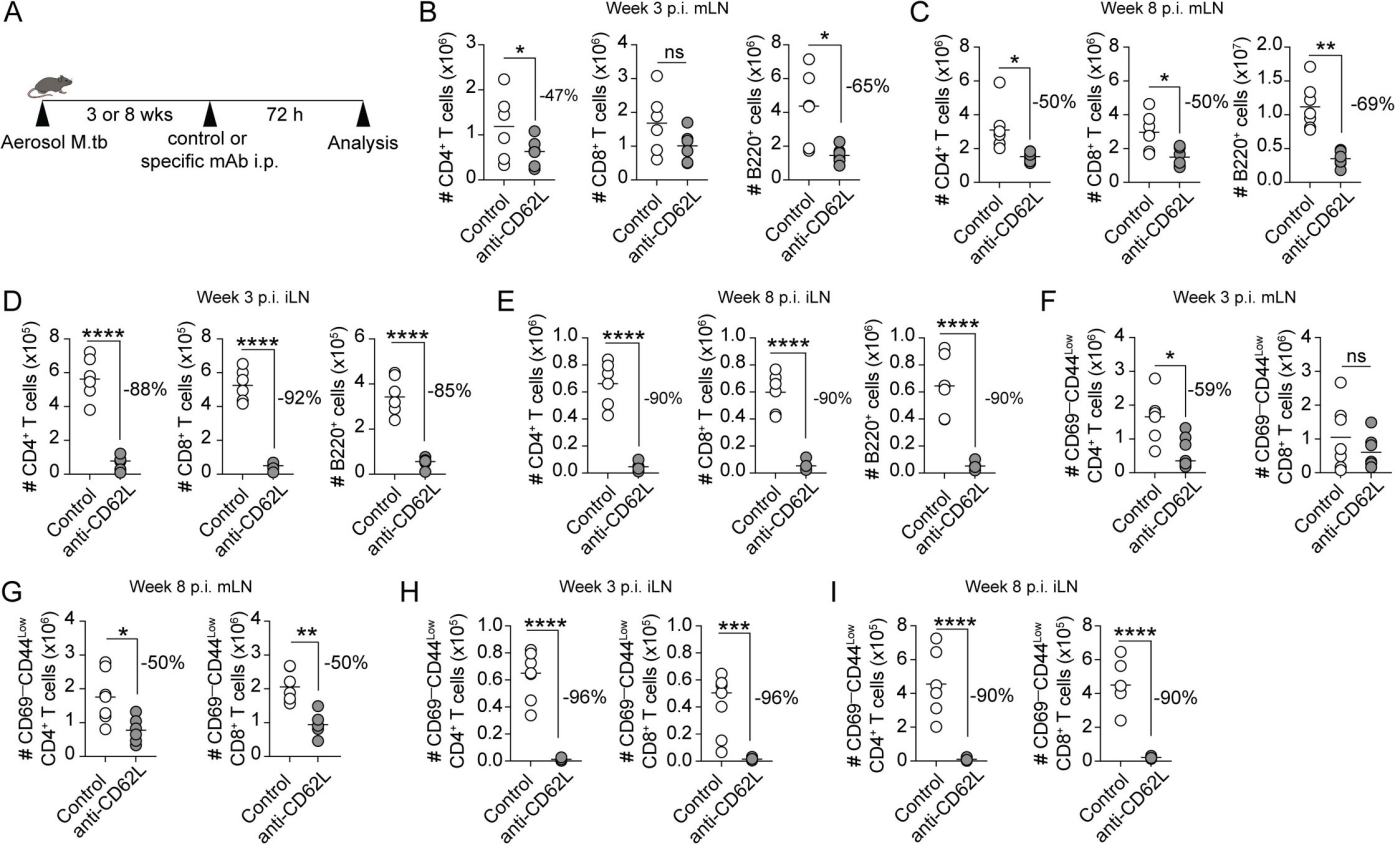
CD62L blockade only partially reduces the homing of lymphocytes into the mLN. (**A**) Experimental design. M.tb-infected mice were injected with an isotype control or anti-CD62L mAb i.p. at wk 3 or 8 p.i. After 72 h iLN and mLN were collected for analysis. Number of endogenous CD4^+^ and CD8^+^ T cell and B220^+^ B cells in (**B**) mLN week 3 p.i., (**C**) mLN week 8 p.i., (**D**) iLN week 3 p.i., and (**E**) iLN at week 8 p.i. of isotype control and anti-CD62L treated mice. (**F**) Number of CD69^–^CD44^–^CD4^+^ and CD8^+^ T cells in mLN of control and anti-CD62L treated mice at week 3 p.i. and (**G**) week 8 p.i. (**H**) Number of CD69^–^CD44^–^CD4^+^ and CD8^+^ T cells in iLN at week 3 p.i. and (**I**) week 8 p.i. Data shown are pooled from two independent experiments (n = 3–4 mice/experiment). Statistical differences between groups were determined using Student’s *t* test *p < 0.05, ** *p* < 0.01, ***p < 0.001, **** *p* < 0.0001.

The reduced lymphocyte entry from the circulation accompanied by the increased cellularity of mLN raised the possibility of an alternative entry pathway for naïve lymphocytes. To determine whether lymphocytes rely on L-selectin for recruitment to the mLN, we assessed the cellularity of the LN after blocking L-selectin, an essential adhesion molecule for recirculation via HEV [27,28]. A single injection of anti-CD62L antibody (Mel-14) has been shown to prevent trafficking of naïve lymphocytes and in turn reduce LN cellularity by 90% within 3 days post injection [29,30]. We therefore examined the cellularity of the mLN and iLN in 3- or 8-wk M.tb-infected mice at 72 h after anti-CD62L injection (Fig 2A). Interestingly, we found a partial reduction in lymphocytes at week 3 p.i. in the mLN with a 47% reduction in CD4^+^ T cells, no significant decrease in CD8^+^ T cells, and a 65% reduction in the B cell population (Fig 2B). A similar trend of partial reduction was also found in the mLN at week 8 p.i., with 50% reduction in T lymphocytes, and 69% reduction in the B cell population (Fig 2C).

Similar reductions were observed when naïve CD4^+^ and CD8^+^ T lymphocyte populations were identified as CD69^low^ CD44^low^ at 3- and 8-week p.i. (Fig 2F and 2G). Although reduction of naïve CD8^+^ T cell population following L-selectin treatment was not statistically significant at week 3 p.i. (Fig 2F). Nevertheless, these data indicate that L-selectin treatment only partially reduces lymphocyte accumulation in the mLN during early and late stage of M.tb infection. In contrast, we found a 85–96% reduction in the accumulation of total and naïve lymphocytes in iLN following inhibition of L-selectin at 3-and 8-week p.i. (Fig 2D and 2E and 2H and 2I). These findings suggest that a large proportion of naïve lymphocytes enter the mLN from an L-selectin independent pathway during M.tb infection.

**Fig 3:**
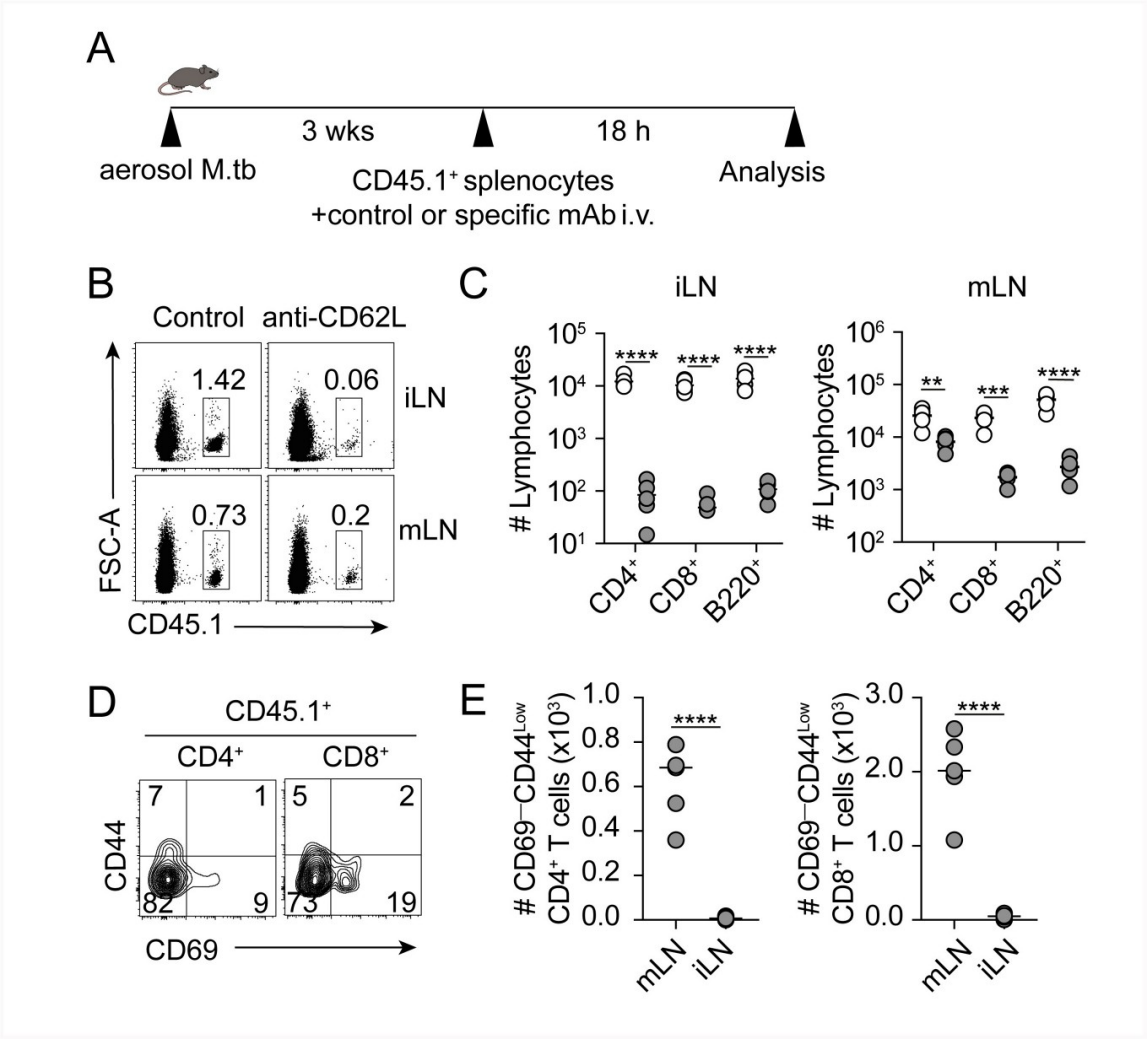
Intravenously injected naïve lymphocytes can enter the mLN via an L-selectin independent mechanism. (**A**) Experimental design. Splenocytes from CD45.1^+^ mice were injected i.v. with isotype control or anti-CD62L mAb into 3wk M.tb-infected CD45.2^+^ mice. After 18 h mLN and iLN were collected for analysis. (**B**) Representative flow cytometry plots showing percentage of donor CD45.1^+^ cells in iLN and mLN of isotype control and anti-CD62L treated mice. (**C**) Total number of CD45.1^+^ CD4^+^ and CD8^+^ T cell and B220^+^ B cells in iLN and mLN of the treated mice. (**D**) Representative flow cytometry plot showing percentage of CD45.1^+^ CD69^–^CD44^–^ naïve CD4^+^ and CD8^+^ T cells in mLN of anti-CD62L-treated M.tb-infected mice. (**E**) Total number of CD45.1^+^ naïve CD4^+^ and CD8^+^ T cells in mLN and iLN of anti-CD62L-treated M.tb-infected treated mice. White circles in (**C, E**) denote mice that received isotype control mAb and grey circles denote mice that received anti-CD62L mAb. Each circle represents an individual mouse. Data in (**B-E**) representative of two independent experiments with similar results (n = 4–5 mice/group). Statistical differences between groups were determined using (**C**) Two-way ANOVA with Sidak’s multiple comparison test (**E**) Student’s *t* test. ** *p* < 0.01, ***p < 0.001, **** *p* < 0.0001.

To address whether lymphocytes from circulation can home into the lymph node independently of L-selectin through an alternative route, such as the afferent lymphatic vessels (LV) draining the infected lungs, we intravenously injected labelled lymphocytes together with anti-CD62L antibody and analyzed lymphocyte populations in the mLN and iLN 18 h post cell transfer (Fig 3A). This 18 h time point was chosen as this is the period required for circulating naïve lymphocytes to traverse the lung [11]. We found that transferred lymphocytes were able to reach the mLN independently of L-selectin, while they failed to enter the iLN (Fig 3B and 3C). Examining further the phenotype of lymphocytes that reached the mLN, we observed that the majority of transferred lymphocytes that reached the mLN using this alternative L-selectin-independent pathway were naïve CD69^low^ CD44^low^ CD4^+^ T cells and CD8^+^ T cells (Figs 3D and 3E and S1A). These results indicate that while the iLN mainly relies on entry via HEV for naïve lymphocyte recruitment, the mLN in M.tb-infected mice can use an L-selectin independent pathway, which is likely via the afferent LV that drain from the lung.

### Naïve lymphocytes migrate from M.tb-infected lungs to draining mLN independently of L-selectin

**Fig 4:**
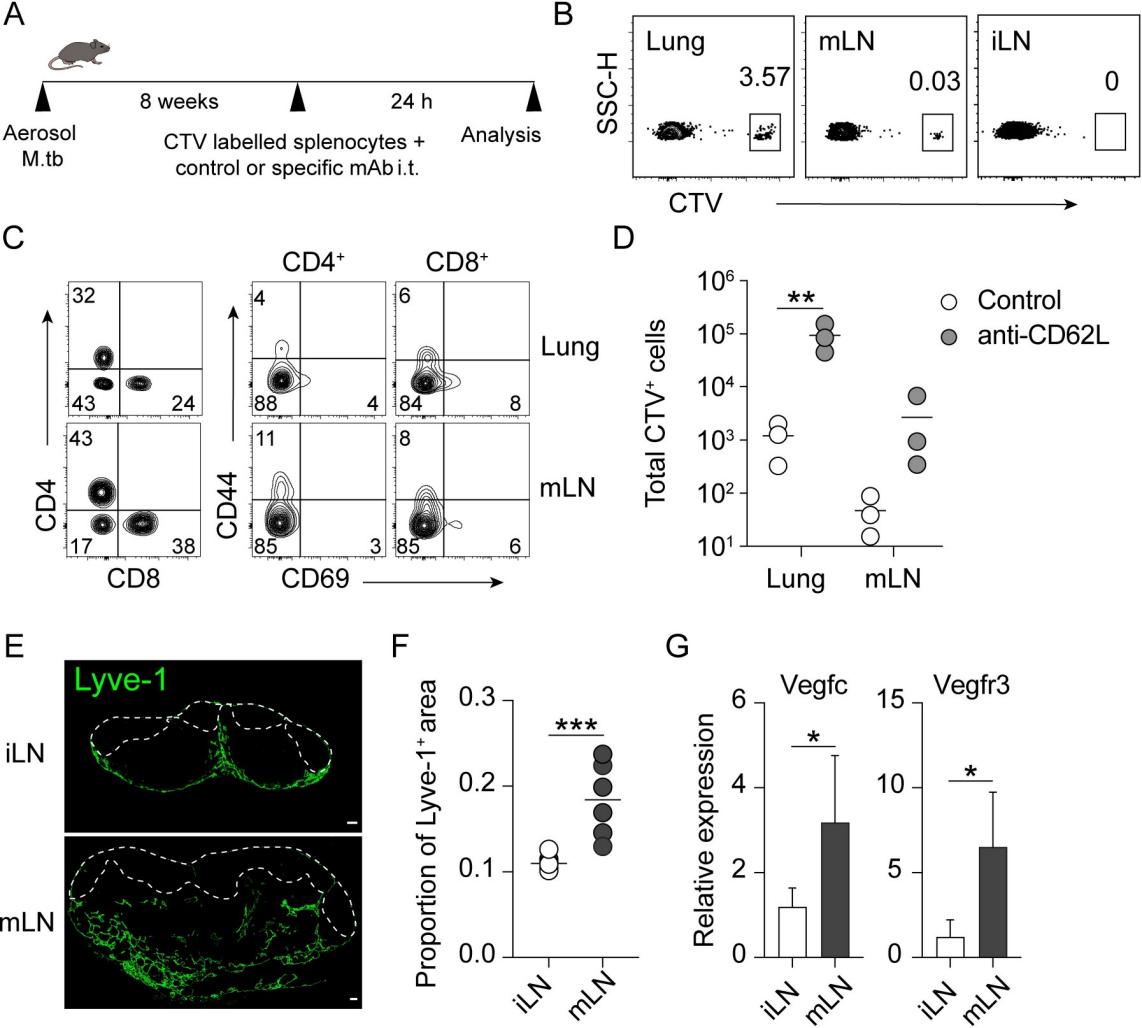
Intratracheally delivered naïve lymphocytes can home to the mLN. (**A**) Experimental design. CTV-labelled lymphocytes were co-injected with isotype control or anti-CD62L mAb i.t. into 8wk M.tb-infected mice. (**B**) Representative flow cytometry plots showing the percentage of CTV^+^ donor lymphocytes in the lung, mLN and iLN. (**C**) Representative flow cytometry plots showing the expression of CD69 and CD44 on CTV^+^ CD4^+^ or CD8^+^ T cells in lung and mLN of 8wk M.tb infected mice is shown. (**D**) Number of CTV^+^ lymphocytes in the lung and mLN of control and anti-CD62L mAb treated M.tb-infected mice. (**E**) Representative immunofluorescence images of Lyve-1 (green) in iLN and mLN. The dashed line delineates the B cell follicles. Scale bar 100 μm. (**F**) Proportion of Lyve-1^+^ region across entire area of the iLN and mLN of M.tb infected mice. Each symbol denotes an individual LN section (2 sections/mouse). (**G**) Expression of *vegfc* and *vegfr3* in iLN and mLN from 8wk M.tb-infected mice determined using quantitative RT-PCR. White symbols in (**D**) denote mice that received isotype control mAb and grey symbols denote mice that received anti-CD62L mAb. Data shown are the mean fold increase ±SD relative to iLN. Data shown are representative of two independent experiments with similar results (3 mice/group). Statistical differences between groups were determined using (**D**) Two-way ANOVA with Sidak’s multiple comparison test and (**F, G**) Student’s *t* test. *p < 0.05, ** *p* < 0.01, ***p < 0.001.

To determine directly whether naïve lymphocyte can exit M.tb-infected lungs and migrate to the mLN via the LV, we transferred CTV-labelled lymphocytes intratracheally into M.tb-infected mice. In additional, to exclude the possibility of intratracheally delivered cells entering the mLN from the blood, these cells were injected together with anti-CD62L mAb (Fig 4A). Intratracheally administered lymphocytes were then analysed 24h post-transfer, a timeframe allowing accumulation of the injected lymphocyte in the mLN (Fig 4A). The greatest frequencies and numbers of labelled lymphocytes were identified in the lungs, followed by the mLN, and no cells were detected in the iLN in both isotype control and anti-CD62L antibody-treated mice (Fig 4B and 4D). Phenotypic analysis of T cells recovered from the lung and mLN showed that they were mainly naïve lymphocytes (CD69^low^ CD44^low^) (Fig 4C). Consistent with previous studies [31], there was an increase in the number of cells recovered from the lungs and mLN in the group that received lymphocytes together with anti-CD62L antibody.

Given the recruitment of lymphocytes from the lung to mLN via an L-selectin independent pathway in M.tb-infected mice, we examined the LV network in iLN and mLN of M.tb-infected mice. As expected, LV structure, identified by Lyve-1 marker, were mainly restricted to the capsule and medullary region of iLN (Fig 4E). In contrast, the LV network was markedly altered in mLN of the infected mice so that Lyve-1^+^ LV cells were no longer restricted to the capsule and medullary area but extended deeper into paracortical region. We also observed an increased expansion of the area occupied by LV within the mLN compared to the iLN (Fig 4F). The expansion of LV network was associated with an increase in the expression of lymphangiogenic factors, VEGF-C and its receptor VEGFR3, in mLN compared to iLN in the infected mice (Fig 4G). These findings collectively indicate that naïve lymphocytes can enter from the lung into the mLN of M.tb-infected mice.

### Inhibiting lymphangiogenesis reduces the entry of naïve lymphocytes to the mLN

**Fig 5:**
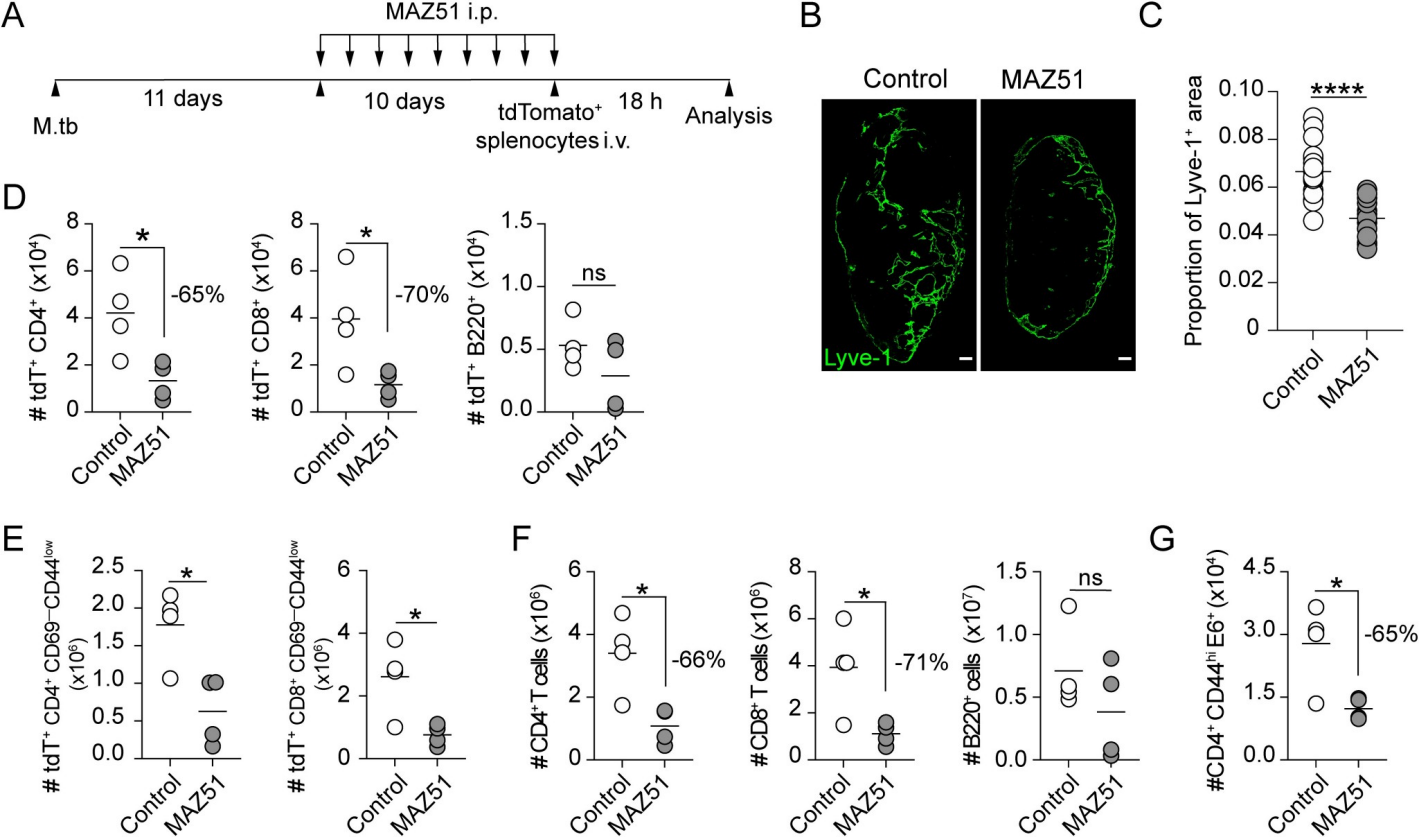
Inhibiting lymphangiogenesis reduces T cell recruitment to the mLN. (**A**) Experimental design. M.tb-infected mice were treated with MAZ51 i.p daily for 10 days, starting from day 11 post aerosol M.tb infection. TdTomato^+^ splenocytes were transferred 18 h before harvest. (**B**) Representative images of Lyve-1^+^ (green) in MAZ51- and vehicle-treated control mice (scale bar, 150 μm). (**C**) Proportion of Lyve-1^+^ region across entire area of MAZ51- and vehicle-treated control mice. Each symbol denotes an individual LN section (4–5 sections/mouse). (**D**) Total number of tdTomato^+^CD4^+^, CD8^+^ and B220^+^ lymphocytes in mLN of control and treated mice. (**E**) Total number of tdTomato^+^ CD69^-^CD44^low^ naïve CD4^+^ and CD8^+^ T cells in mLN of control and MAZ51-treated mice p.i. (**F**) Number of total endogenous CD4^+^, CD8^+^ and B220^+^ lymphocytes in mLN of control and MAZ51-treated mice. (**G**) Number of endogenous ESAT6_4-17_ specific (ESAT6_4-17_:I-A^b^) CD4^+^ T cells in mLN of control and MAZ51 treated mice. Each symbol represents an individual animal. Statistical differences between groups were determined using Student’s *t* test. *p < 0.05, **** *p* < 0.0001.

To examine the role of afferent lymphatics in lymphocyte transmigration, M.tb-infected mice were injected daily with a VEGFR3 kinase inhibitor, MAZ51, starting from 2 weeks post-M.tb infection. This inhibitor has been demonstrated to be effective in suppressing lymphangiogenesis in mice [32]. Ten days after MAZ51 treatment, the mice received a transfer of tdTomato^+^ lymphocytes i.v. from naïve donor mice, and the transferred cells were tracked 18 hours later (Fig 5A). To determine the efficacy of this inhibitor in our model, we examined LV expansion in the mLNs of vehicle and MAZ51 treated mice (Fig 5B and 5C). We found that MAZ51 treatment resulted in a significant reduction in Lyve-1^+^ area compared to vehicle- treated control mice (Fig 5C). We also observed that, compared to vehicle control, MAZ51 treatment led to a significantly reduced recruitment of the total tdTomato^+^ CD4^+^ and CD8^+^ T cells (65% and 70%, respectively) (Fig 5D), as well as naïve CD69^low^ CD44^low^ tdTomato^+^ CD4^+^ and CD8^+^ T cells (60% for both subsets) (Fig 5E), into the mLN. Interestingly, the B cell population was not significantly decreased (Fig 5D), which is consistent with our previous results (Figs 2B and 2C and 3C) that showed only a small number of B cells entering the mLN via the L-selectin-independent pathway.

A similar pattern of reduced T cell entry was also observed for endogenous cells, where a significant reduction in CD4^+^ and CD8^+^ T cells (66% and 71%, respectively), but not in the number B lymphocytes (Fig 5F). Importantly, blockade of VEGFR3 resulted in 56% reduction in the numbers of endogenous tetramer-positive ESAT-6_4−17_-specific CD4^+^ T cells in mLN (Fig 5G). The numbers of endogenous or exogenously injected (tdTomato^+^) lymphocytes, as well as tetramer-positive ESAT-6_4−17_- specific CD4^+^ T cells, did not differ in the lungs of MAZ51- and vehicle-treated mice (S2A Fig). Interestingly, we found a decrease in the frequency of IFN-γ, TNF-α, but not IL-2, expressing CD4^+^ T cells in the lungs of MAZ51-treated mice compared to vehicle-treated controls mice (S2B Fig). However, this could be due to reduced output of effector cells from mLN to the lungs, as effector T cells utilize the efferent LV as an exit route [4,33]. We also found that the bacterial burden in mLN and the lungs were also comparable regardless of the treatment (S2C Fig). We could not exclude the possibility that the minimal effect of MAZ51 treatment on the dynamics of pathogen-host interaction in the lungs and mLN is due to the short treatment duration used. Nonetheless, these data indicate that afferent lymphatic vessels contribute to the entry of naïve T lymphocytes to the mLN during M.tb infection.

### Ag-specific CD4^+^ T cells entering in mLN independent of L-selectin are activated locally to become Th1 cells

**Fig 6:**
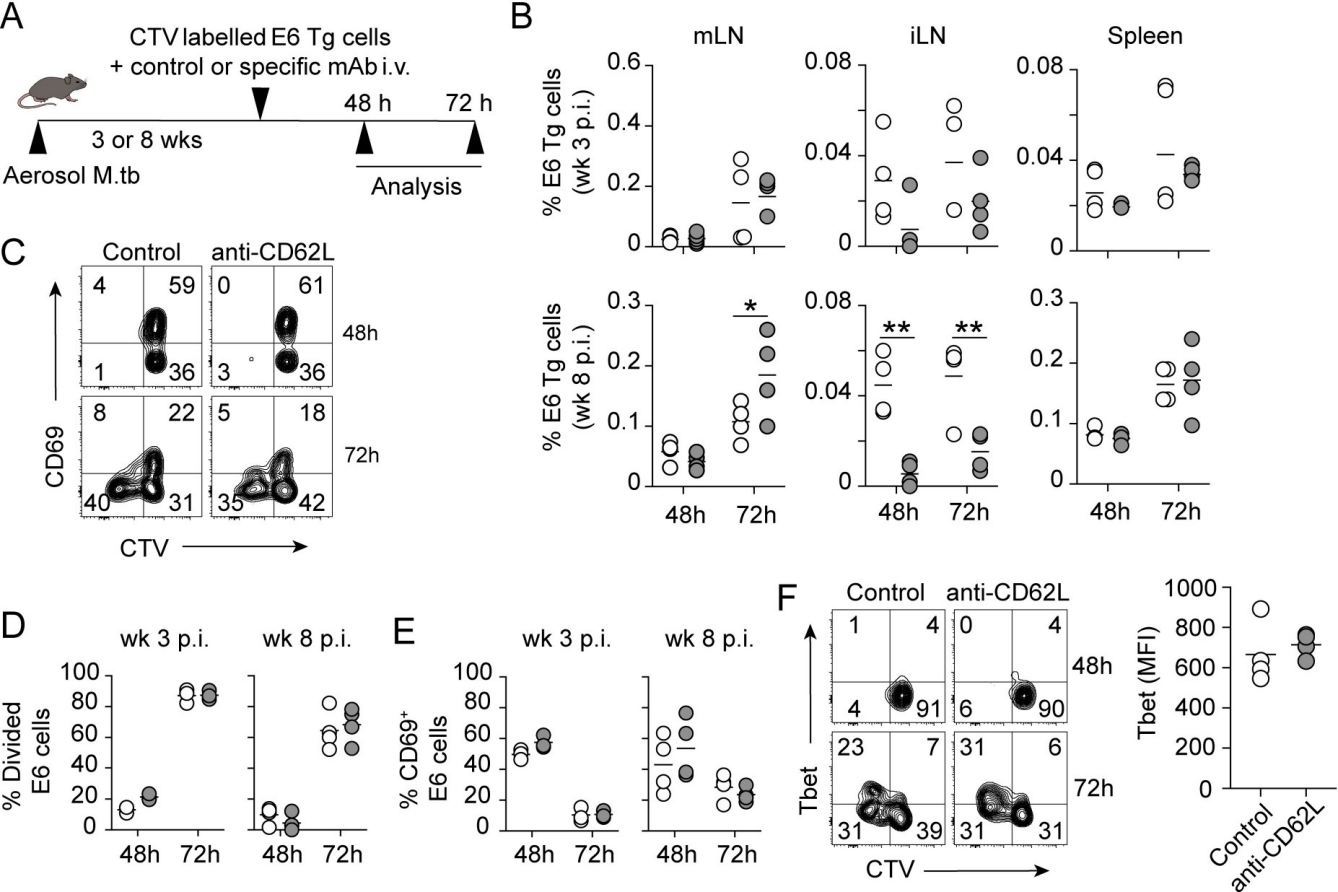
Ag specific CD4^+^ T cells entering in an L-selectin independent pathway are activated in the mLN. (**A**) Experimental design. Splenocytes from C7.GFP.*Rag1*^*—/—*^Tg mice were CTV labelled and injected i.v. into 8wk M.tb-infected WT recipients (∼ 2 x 10^5^ CD4^+^ transgenic T cell / mouse). The mLN, iLN and spleens were analyzed at 48 h and 72 h post-transfer. (**B**) Percentage of E6 Tg cells in mLN, iLN and spleen of control and anti-CD62L mAb treated mice at 48 h and 72 h post cell transfer at week 3 p.i. and week 8 p.i. (**C**) Representative flow cytometry plots showing the percentage of CD69^+^ as well as CTV^high^ and CTV^low^ E6 Tg cells at 48 h and 72 h post transfer in mLN of control and anti-CD62L treated mice at week8 p.i.. (**D**) Summary graph of the proportion of divided E6 Tg cells (CTV ^low^) and proportion of activated CD69^+^ E6 Tg cells in mLN of the treated mice at week 3 and 8 p.i. (**E**) Representative flow cytometry plots showing the co-expression of T-bet and CTV in E6 Tg cells at 48 h and 72 h post transfer in mLN of the treated mice. Summary graph showing MFI of T-bet in E6 Tg cells at 72 h post-transfer in control and treated mice at week 8 p.i. Data shown are representative of two independent experiments with similar results (n = 4 mice/group). White circles denote mice that received isotype control mAb and grey circles denote mice that received anti-CD62L mAb. Statistical differences between groups were determined by (**B, D and E**) Two-way ANOVA with Sidak’s multiple comparison test or (**F**) Student’s *t* test *p < 0.05, ** *p* < 0.01.

To determine whether lymphocytes entering via an L-selectin independent pathway are antigen responsive, we adoptively transferred GFP-expressing, ESAT6_1-20_ epitope-specific TCR transgenic CD4^+^ T cells (Clone 7) together with anti-CD62L mAb or isotype control into 3 or 8 week M.tb infected recipient mice, and donor cells (GFP^+^) were analysed at 48 h and 72 h post-transfer (Figs 6A and S1B). Interestingly, flow cytometric analysis of mLN showed no difference in the frequency of E6 Tg cells in the mLN at 48 h between control and anti-CD62L-treated LN, however, we saw an increase at 72 h in the anti-CD62L-treated group (Fig 6B). As anticipated no difference in the frequency of E6 Tg were detected in the spleen between anti-CD62L-treated and control groups at week 3 and 8 p.i., as lymphocyte recruitment in this secondary lymphoid organ is L-selectin independent [31]. In corroboration with our earlier results, a significant decrease was found in the frequency of E6 Tg cells in iLN that received L-selectin compared to isotype control (Fig 6B). These data reaffirm our previous results, suggesting that lymphocytes can reach the mLN in L-selectin-independent pathway.

We next analysed the response of CTV-labelled E6-specific CD4^+^ T cells in the mLN by comparing the rate of cell division and expression of the early activation marker CD69 and Th1-specific transcription factor T-bet in anti-CD62L or isotype control mAb treated M.tb-infected mice at week 3 and 8 p.i. (Fig 6C–6E). We found that cell division, as measured by CTV dilution, began between 48 and 72 h post cell transfer, and the percentage of CTV^Low^ E6-specific T cells was comparable between the two-treatment groups at both weeks 3 and 8 p.i. (Fig 6C and 6D). CD69 expression was upregulated at 48 h post cell transfer and began to decrease by 72 h at the beginning of cell division regardless of treatment (Fig 6C and 6D). Suggesting that L-selectin independent pathway provides antigen responsive cells in early and late stage of M.tb infection. Similarly, the kinetics of T-bet upregulation was equivalent between anti-CD62L and isotype control treated groups (Fig 6E). Finally, although we found an increase in total lymphocyte numbers and tetramer-positive ESAT-6_4−17_ CD4^+^ T cells in the lungs after 72 h of L-selectin treatment compared to isotype control (S3A Fig), we observed no difference in the number of tetramer-positive Ag85b CD4^+^ T cells (S3A Fig), or in the frequency of IFN-γ, TNF-α and IL-2 expressing pulmonary CD4^+^ T cells following restimulation with ESAT6 antigen *ex vivo* (S3B Fig). This could be due to the short-term treatment with anti-CD62L, as effector cells are likely to be already in the lungs after 3 weeks p.i. [21]. Taken together, these data indicate that the L-selectin-independent pathway is a physiologically functional route for supplying antigen responsive cells to the mLN during M.tb infection.

## Discussion

A pivotal step in the initiation of the adaptive immune response is the recruitment of naïve Ag-specific lymphocytes to LN draining a site of infection or immunization. In this study, we showed that during pulmonary M.tb infection, the entry of naïve lymphocytes into the lung-draining LN via the L-selectin dependent HEV pathway was significantly impaired compared to entry into non-draining LN. Approximately half of the naïve T cells entered the mLN via a L-selectin-independent mechanism. We further showed that persistent M.tb infection promotes lymphangiogenesis and the afferent LVs play a previously unappreciated role in routing circulating naïve T cells to the mLN through the lung. These findings reveal that the L-selectin-independent mechanism controls the entry and priming of naïve T lymphocyte in the mLN during M.tb infection, and we propose that this alternative route of entry is important in sustaining adaptive immune responses to persistent pulmonary infection.

HEV have been well documented to be the main gateway for the homing of naïve lymphocyte from the circulation to LN [4,34,35]. Most studies examining changes in lymphocyte recirculation during infection or immunization have reported an increase in lymphocyte recruitment to the draining LN via HEV [12,36,37]. This enhanced recruitment is associated with an efficient initiation of the adaptive immune response, as it ensures the increase in antigen-specific T cell pool to support the ongoing immune response [12]. Unexpectedly, we found that homing of circulating naïve lymphocytes via HEV is reduced in mLN during M.tb infection. This reduced recruitment was associated with decreased expansion of HEV along with disrupted expression of the homing chemokine CCL21 in the mLN. Similar alterations in the lymphocyte homing machinery have been previously shown to lead to LN atrophy during infection with a pathogenic strain of chikungunya virus (AF15561) [17]. By contrast, we observed that the cellularity of mLN increased strikingly following M.tb infection, indicating that homing via HEV is unlikely to account solely for the accumulation of lymphocytes in mLN.

In addition to HEV, the afferent LV is a secondary portal for leukocyte recruitment into the LN. Unlike the HEV pathway, homing of lymphocytes via the afferent LV has long been considered to be a minor contributor to LN cellularity [9,33]. The current concept on naïve lymphocyte trafficking was largely based on cannulation studies in sheep or adoptive transfer experiments performed in mice [10,38–40], in which inflammation promoted the migration of APCs and effector lymphocytes into the draining LN predominantly via the afferent LV. However, it is unclear whether these distinct trafficking patterns for naïve and effector lymphocytes are similar for LN draining other organs during infection. Previous studies have suggested that low numbers of naïve lymphocytes are known to circulate through highly vascularized non-lymphoid organs, such as the lung [11,41] and the liver [31]. It has also been demonstrated that following M.tb infection CD4^+^ T lymphocytes accumulate in lung parenchyma, peaking around week 3–4 p.i. and persisting until at least 6 months p.i. [41]. Naïve CD4^+^ T cells were further shown to be more efficient at entering the parenchyma from the blood vasculature than effector cells. The transit of naïve lymphocytes into the lung parenchyma suggests the possibility that these cells can exit the lung and traffic to the mLN through the LV, and this has indeed been proposed to occur in naïve mice [11]. In this study, we found that a substantial proportion of naïve lymphocytes are capable of homing to the mLN even when L-selectin is blocked, which suggests that these naïve cells are likely entering into the mLN via the afferent LV. In addition to the contribution of the L-selectin-independent pathway to lymphocyte homing to the mLN at the peak of adaptive immunity (week 3) and the chronic phase (week 8), as demonstrated in this study, it would be interesting to investigate in the future the impact of L-selectin blockade and characterize early recirculation events at the onset of adaptive immunity in the mLN. This investigation could provide new insights into the delay in the generation of adaptive immunity following aerosol M.tb infection.

By using two distinct approaches, we were able to confirm that the naïve T lymphocytes that entered the mLN in the absence of L-selectin-HEV interaction were migrating from circulation via the lung-draining LV. First, we found that the LV network underwent extensive expansion in the lung-draining mLN, but not in distant LN draining a non-infection site. Through the inhibition of lymphangiogenic factor VEGFR3, we confirmed an essential role for the M.tb-induced lymphangiogenesis in the recruitment of both naïve and antigen-specific T lymphocytes to the mLN during M.tb infection. Second, by intratracheal transfer of lymphocytes we demonstrated that naïve lymphocytes could exit the lung and home into the mLN in an L-selectin-independent manner. Our finding is consistent with recent studies performed in a different respiratory disease model [42,43], establishing a critical role for lung-draining LV in supplying naïve lymphocytes to LN. Thus, under conditions where direct naïve T cell entry from blood circulation is impaired, as demonstrated in this study, T cell accumulation in the lung and the subsequent trafficking into the mLN can act as an alternative pathway to compensate for this defect. It would therefore be interesting to examine whether this alternative route is active during other respiratory pathogens, and assess its contribution to the supply of naïve lymphocytes to the mLN. In the future, it will also be important to investigate whether vaccination before M.tb challenge would modify the entry of naïve lymphocytes via the lung, and whether this L-selectin independent mechanism could be exploited in vaccine development strategies. It will also be interesting to investigate whether similar mechanism operates in other large, vascularized organs, such as the liver. Both the lung and liver are large solid organs with very limited numbers of draining lymph nodes in mice [44,45], and mechanisms that permit entry of naïve lymphocytes via alternative routes may facilitate the immune surveillance of these organs by ensuring adequate numbers of naïve lymphocytes circulating through LN.

We further demonstrate that the L-selectin-independent homing mechanism is functional in M.tb infection and contributes to the generation of Ag-specific effector T cells in the mLN. The kinetics of CD4^+^ T cell activation and differentiation into effector Th1 cells were comparable between isotype control and anti-CD62L antibody-treated mice. Interestingly, it has been previously reported in a mouse model of M.tb infection [46] that the frequency of IFN-γ-producing CD4^+^ T cells quantified following *in vitro* restimulation with plate bound CD3/CD28, were decreased in the mLN of selectin ligand deficient FtDKO mice. These mice lack the expression of fucosyltransferases IV and VII, which prevents the binding of both naïve lymphocytes via L-selectin ligand and activated T cells with E and P-selectin ligands [47]. Therefore, the observed reduction in IFN-γ-producing CD4^+^ T cells could be due to impaired recruitment of naïve T lymphocytes and/or activated T cells to the mLN. However, our study is consistent with recent findings in a mouse model of influenza virus infection, where no changes in the activation of CD8^+^ T cells in the mLN of L-selectin deficient and anti-L-selectin antibody treated mice was observed [42]. All together these data indicate that the L-selectin independent pathway can act as a unique complementary pathway for recruiting a pool of naïve lymphocytes to ensure the efficient supply of antigen-responsive CD4^+^ T cells to the mLN during M.tb infection.

While the generation of effector CD4^+^ T cells is known to restrict M.tb infection, the pathogen survives in the face of host adaptive immunity leading to persistent infection [21]. Mechanisms regulating this host-pathogen equilibrium are incompletely understood. Our study reveals an alternative homing route for supplying naïve lymphocytes to the lung draining LN in murine TB. This mechanism may be particularly important in the setting of chronic respiratory infection where L-selectin-dependent pathway is restricted due to local inflammation. Understanding the regulatory mechanisms governing naïve T cell trafficking in chronic infection and how these may differ between different organs may ultimately help the development of more specific, host-directed therapies for pathogen elimination.

## Materials and methods

### Ethics approval

All murine experiments were conducted with the approval of the Sydney Local Health District Animal Welfare Committee (protocols 2015–037 and 2020–007).

### Mice

CD45.2^+^ and CD45.1^+^ C57BL/6 (6–8 weeks of age) were purchased from Australian BioResources (Moss Vale, NSW, Australia). TCR transgenic (Tg) mice specific for ESAT6_1-20_-IA^b^ complexes (Clone 7 (C7)) were bred with GFP-expressing B6 *Rag1*^*—/—*^mice to generate E6 [48]. GFP^+^.*Rag1*^*—/—*^[49] and tdtomato^+^ mice that were bred and maintained at the Centenary Institute Animal Facility.

### M.tb culture and infection

M.tb (strain H37Rv) was grown to log phase at 37 °C in Middlebrook 7H9 broth (BD Biosciences), supplemented with 0.5% (v/v) glycerol, and 0.05% (v/v) Tween 80 (Sigma Aldrich) and 10% albumin-dextrose-catalase. Mice were infected with H37Rv via aerosol route using a Middlebrook airborne infection apparatus (Glas-Col) with an infective dose of ∼100 viable bacilli per lung. Colonies forming units (CFU) in tissue homogenates were quantified using Middlebrook 7H11 Agar (BD Biosciences) supplemented with oleic acid-albumin dextrose catalase and 0.5% glycerol.

### Treatments with anti-CD62L and VEGFR3 kinase inhibitor

For blockade of CD62L, mice received a single dose (250 μg/mouse Mel-14; BioXcell) intraperitoneally at weeks 3 or 8 p.i. Mice were culled 72 h after anti-CD62L treatment for analysis. For VEGFR3 kinase blockade mice received daily doses of MAZ51 (10 mg/kg/day; Calbiochem) intraperitoneally for 10 days before harvest at week 3 p.i.

### Adoptive cell transfer

Splenocytes from CD45.2^+^, CD45.1^+^, tdtomato^+^ or E6 mice were collected and processed to single cell suspensions. CD45.2^+^ and E6 cells were labelled with 5 μM CellTrace Violet (CTV) (Thermo Fisher Scientific) in PBS supplemented with 0.1% FCS for 20 min in the dark at 37°C. CTV labelling was terminated by adding cold RPMI media supplemented with 10% FCS. All cell suspensions were washed and resuspended in PBS prior to cell transfer. Where indicated, ∼2x10^7^ CTV labelled CD45.2^+^, CD45.1^+^ or tdTomato^+^ cell or ∼2x10^6^ E6 TCR Tg cells (containing ∼2 x 10^5^ CD4^+^ T cells) were injected intravenously (i.v.) into CD45.2^+^ mice. Where indicated, mice received cell transfer (i.v.) with anti-CD62L (250 μg/mouse Mel-14; BioXcell) or rat IgG2a isotype control (250 μg/mouse; BioXcell).

### Preparation of single suspension from tissues

Lymph nodes, spleens, and lungs were collected in RPMI supplemented with 2% foetal calf serum (FCS) for flow cytometric analysis. LN and spleens were mechanically dissociated through 70 μm strainers. Lungs were perfused with PBS by cardiac cannulation and collected in 2 mL RPMI media supplemented with 0.1 mg/mL DNase I (Sigma Aldrich) and 10 U/mL collagenase type I (Sigma Aldrich). Lungs were dissociated using GentleMacs Dissociater (Miltenyi Biotec) and incubated for 30 min at 37°C. Lungs were then further dissociated with GentleMacs Dissociater and filtered through a 40 μm strainer. Single cell suspensions were treated with ACK lysis buffer to remove erythrocytes. Cells were washed with RPMI media supplemented with 2% FCS before viable cells were counted using trypan blue exclusion on a hemocytometer.

### Flow cytometry

Spleens, LN and lungs were stained in FACS wash (2%FCS/PBS/2mM EDTA) containing surface receptor antibody mixture, Fc block (2.4G2; BD Biosciences) and Live/Dead fixable blue dead cell stain (Thermo Fisher Scientific) for 30 min at 4°C in the dark. The following antibodies and clones were used for the detection of surface markers: CD4(RM4-5), B220 (RA3-6B2), CD8 (53–6.7) CD44 (IM7), and CD69 (H1.2F3) (all from BD biosciences). For Cytokine expression analysis, ∼5x10^6^ cells from the lungs were stimulated with 10 μg ESAT6_1-20_ peptide (GeneScript) in the presence of 1:1000 Golgi-plug (BD Bioscience) for 6h at 37°C. Samples were then washed in cold RPMI supplemented with 2% FCS. For detection of intracellular molecules surface-stained cells were fixed with 100 μL Cytofix fixation buffer (BD Biosciences) for 15 min at 4°C. Cells were washed in 1x permeabilization buffer (BD biosciences). Cells were incubated for 1 h at 4°C in 1x Permeabilization buffer containing the appropriate antibodies. The following antibodies and clones were used for the detection of intracellular molecules: T-bet (4B10; Thermo Fisher Scientific), IFN-γ (XMG1.2; BD Biosciences), IL-2 (JES6-5H4; BD Biosciences), and TNF-α (MP6-XT22; BD Biosciences). For detection of antigen-specific CD4^+^ T cells, single cell suspensions were incubated in FACS wash (2%FCS/PBS/2mM EDTA) containing ESAT6_4-17_:I-A^b^ and Ag85B_240-254_:I-A^b^ (NIH Tetramer Core Facility, Atlanta, GA, USA) and Fc block (2.4G2; BD Biosciences), for 1 h at 37°C prior to antibody staining of surface markers. Cells were washed in 1x permeabilization buffer and resuspended in FACS buffer prior to acquisition. All flow cytometry data acquisition was performed on LSRII using FACSDiva software (BD Biosciences) and analysis was performed using FlowJo v10.8.0.

### Organ collection and processing for imaging

LN were collected and fixed in 1% paraformaldehyde (Alfa Aesar) at 4°C for 24 hours. Fixed tissues were then placed in 30% (v/v) sucrose (Sigma Aldrich) in PBS. Twenty-four hours later tissues were frozen in Optimal cutting temperature compound (VWR chemicals). Tissues were sectioned at 10–14 μm on a Shandon Cryotome E (ThemoFisher). Sections were blocked and permeabilised with 3% normal goat serum and 0.1% Triton X-100 diluted in PBS (Cell Signaling Technologies) for 30 minutes at room temperature. Sections were then stained with the appropriate primary antibodies diluted in 3% normal goat serum (NGS) in PBS for 2 h at room temperature or overnight at 4°C. Sections were washed in 1x tris-buffered saline with 0.5% tween-20 (TBST) before staining with secondary antibodies diluted 3% NGS in PBS for 1 h at room temperature. The sections were washed 3x in 1x TBST, and subsequently mounted with prolong gold antifade (Thermo Fisher). The following antibodies (clone number; source) were used in this study: CD3 (17A2; BioLegend), B220 (RA3-6B2; BD Biosciences), Lyve-1 (polyclonal ab33682; Abcam), CCL21/6Ckine (polyclonal; R&D Systems), PNAd (MECA79; Biolegend) and CD31 (polyclonal ab124432; Abcam); goat anti-rabbit Alexa-fluor 647 (polyclonal; ThermoFisher) or Alexa-fluor 555 (polyclonal; ThermoFisher), streptavidin Alexa Fluor 555 (BD biosciences). Sections were imaged on a Deltavision Personal (GE Healthcare). Image analysis was performed using FIJI software v2.0.0-rc-69/1.53g (NIH Research Services, Bethesda, MD).

### Image analysis

The number of HEV (MECA79^+^ CD31^+^) were counted on whole LN sections and were normalised to the total area of the LN section. The number of HEV with continuous (CCL21^+^) or intermittent (partial and/or no detection) (CCL21^—^) expression were counted on whole LN sections. All cell counting was performed using the cell counter plugin in Fiji software. The area covered by Lyve-1 was measured in mm^2^ on whole LN sections and was normalised to the total area of the LN sections. The area was measured using Fiji software.

### mRNA preparation and qRT-PCR

LN were collected and submerged in RNAlater (Ambion) for 24 h (4°C) prior to long term storage at -80°C. RNA was purified from lymph nodes using Trisure (Bioline) as per manufacturer’s instructions (Bioline). RNA (2 μg) was reverse transcribed using the Tetro cDNA synthesis kit with random primers according to the manufacturer’s instructions (Bioline). Quantitative reverse transcriptase PCR (qRT-PCR) was performed using SensiFast SYBR Green (Bioline) on a Roche LightCycler480. Data are expressed as fold change over uninfected control lymph nodes. Data were calculated by ΔΔCT method using 18S as the reference gene. Forward and reverse qRT-PCR primers used in this study: *18S* Fw GTAACCCGTTGAACCCCATT, Rv CCATCCAATCGGTAGTAGCG, *vegfr3* Fw GCGACAGGGTTCTCATAA, Rv CGTTGCCTCATTGTGATTAG, *vegfc* Fw CAGCCCACCCTCAATACCAG, Rv GCTGCTCCAAACTCCTTCC.

### Statistical analysis

Statistical analysis was performed using GraphPad Prism 9 software (GraphPad Software, La Jolla, CA). Differences between two groups were analyzed by Student’s *t*-test. Analysis between multiple groups was performed using one-way or two-way analysis of variance (ANOVA) with Tukey’s or Sidak multiple comparisons test respectively. Results with *p* < 0.05 were deemed statistically significant.

## Supporting information

S1 Fig Flow cytometry gating strategy. (**A**) Lymph node samples were gated for total lymphocytes, singles cells, and live cells. Live cells were then gated for donor CD45.1^+^. CD45.1+ were further gated on CD4^+^ and CD8^+^ T cells. The expression of CD44 and CD62L was then identified on either CD4^+^ or CD8^+^ T cells. (**B**) Lymph node samples were gated on total lymphocytes, single cells, and live cells as shown in (**A**). Live cells were then gated on CD4+ and GFP+ to identify donor E6 Tg GFP^+^ CD4^+^ T cells. The expression of CD69 or Tbet against CTV was then analysed on E6 Tg CD4^+^ T cells.
(TIF)

S2 Fig Effect of MAZ51 on lymphocyte recruitment in the lung. (**A**) Total number of endogenous lymphocytes, tdT^+^ lymphocytes, and tetramer^+^ ESAT6_4-17_ specific (ESAT6_4-17_:I-A^b^) specific CD44^hi^ CD4^+^T cells in the lungs of control and MAZ51-treated mice p.i. (**B**) IFN-γ, TNF-α and IL-2 expression in CD4^+^ CD44^+^ T cells in the lung of control and MAZ51-treated mice at wk3 p.i. Lung cells were restimulated *in vitro* with ESAT6_1-20_ peptide. (**C**) Bacterial loads in the lung and mLN of control and MAZ51-treated mice measured at wk 3 p.i. Data shown are representative of two independent experiments with similar results (n = 4 mice/group). Statistical differences between groups were determined by Student’s *t* test. *p < 0.05.
(TIF)

S3 Fig Effect of anti-CD62L blockade on lymphocyte recruitment to the lung. (**A**) Total number of endogenous lymphocytes, CD44^+^ ESAT6_4-17_: I-A^b+^ and Ag85B_240-254_:I-A^b^ CD4^+^ T cells in the lungs of wk8 M.tb-infected mice 72 h post isotype control or anti-CD62L i.p. injection. (**B**) IFN-γ, TNF-α and IL-2 expression in CD4^+^ CD44^+^ T cells in the lung of control and anti-CD62L treated mice 72 h post isotype control or anti-CD62L i.p. injection. Data shown are representative of two independent experiments with similar results (n = 4 mice/group). Statistical differences between groups were determined by Student’s *t* test. *p < 0.05, ** *p* < 0.01.
(TIF)
