# Supplementary figures and images for: L-selectin-dependent and -independent homing of naïve lymphocytes through the lung draining lymph node support T cell response to pulmonary Mycobacterium tuberculosis infection
Source: PLoS Pathog. 2023 Jul 5;19(7):e1011460. doi: 10.1371/journal.ppat.1011460 (PMC10321623; doi:10.1371/journal.ppat.1011460)

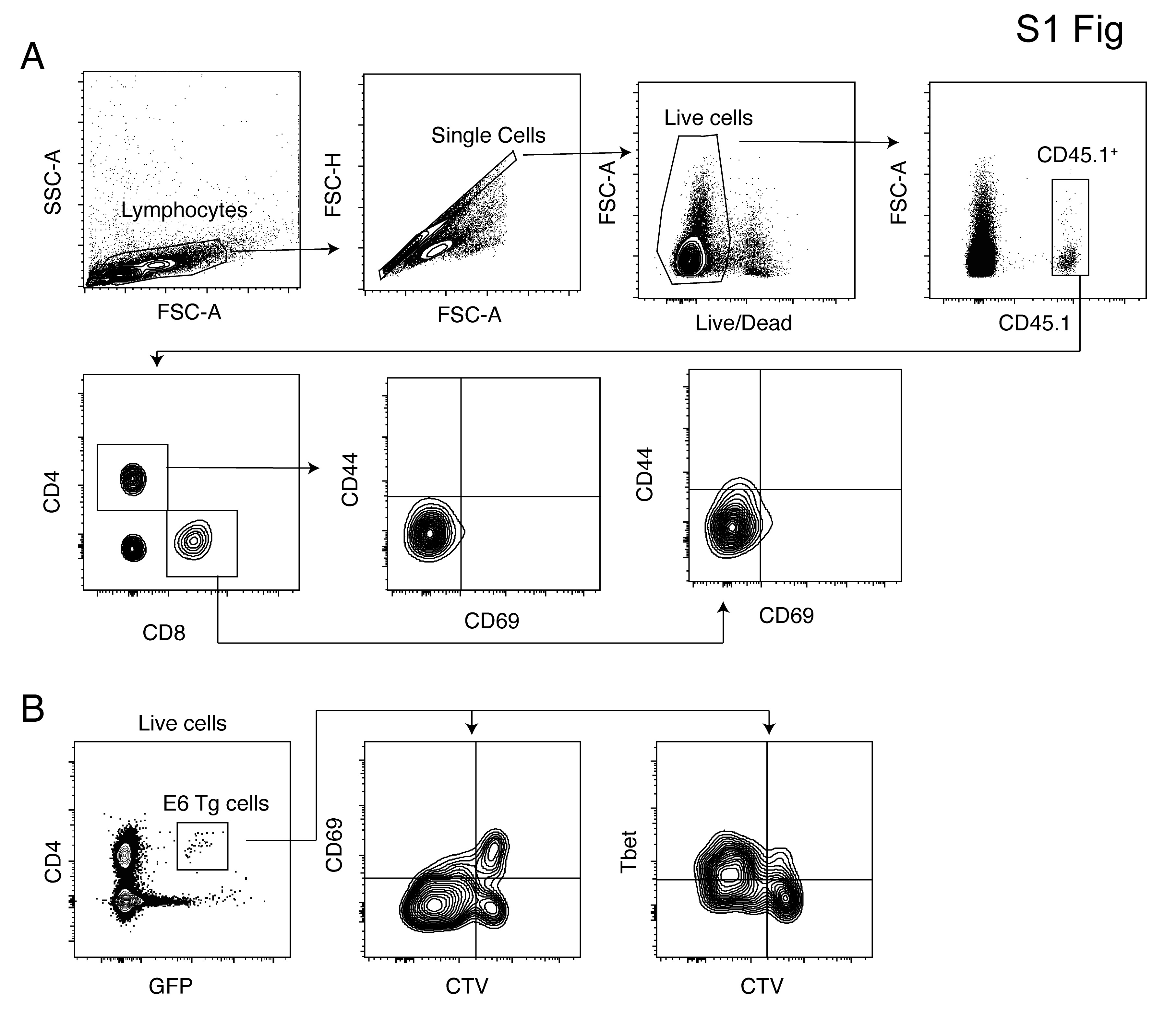

Supplement: S1 Fig — (A) Lymph node samples were gated for total lymphocytes, singles cells, and live cells. Live cells were then gated for donor CD45.1+. CD45.1+ were further gated on CD4+ and CD8+ T cells. The expression of CD44 and CD62L was then identified on either CD4+ or CD8+ T cells. (B) Lymph node samples were gated on total lymphocytes, single cells, and live cells as shown in (A). Live cells were then gated on CD4+ and GFP+ to identify donor E6 Tg GFP+ CD4+ T cells. The expression of CD69 or Tbet against CTV was then analysed on E6 Tg CD4+ T cells. (TIF) [file ppat.1011460.s001.tif]

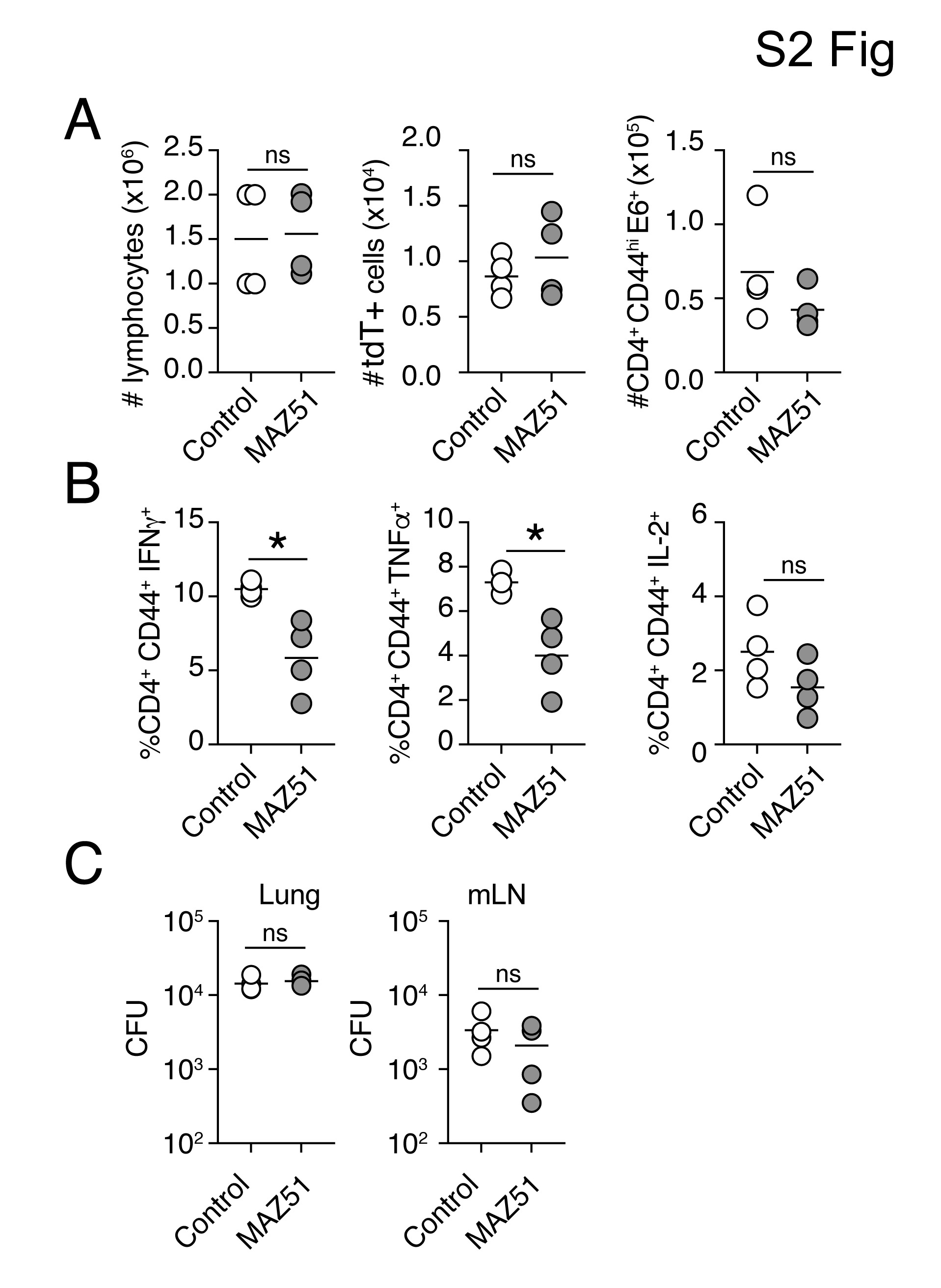

Supplement: S2 Fig — (A) Total number of endogenous lymphocytes, tdT+ lymphocytes, and tetramer+ ESAT64-17 specific (ESAT64-17:I-Ab) specific CD44hi CD4+T cells in the lungs of control and MAZ51-treated mice p.i. (B) IFN-γ, TNF-α and IL-2 expression in CD4+ CD44+ T cells in the lung of control and MAZ51-treated mice at wk3 p.i. Lung cells were restimulated in vitro with ESAT61-20 peptide. (C) Bacterial loads in the lung and mLN of control and MAZ51-treated mice measured at wk 3 p.i. Data shown are representative of two independent experiments with similar results (n = 4 mice/group). Statistical differences between groups were determined by Student’s t test. *p < 0.05. (TIF) [file ppat.1011460.s002.tif]

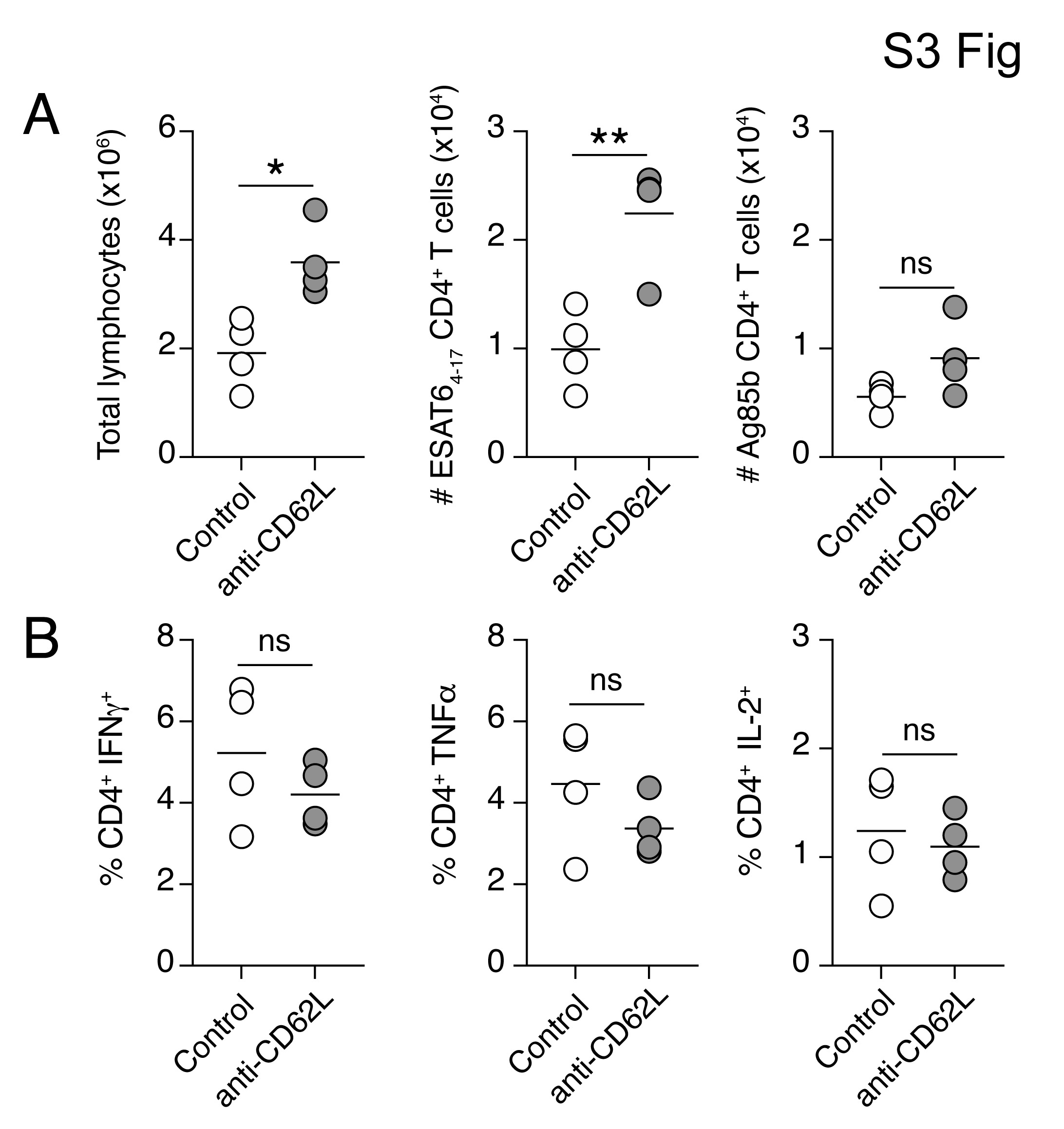

Supplement: S3 Fig — (A) Total number of endogenous lymphocytes, CD44+ ESAT64-17: I-Ab+ and Ag85B240-254:I-Ab CD4+ T cells in the lungs of wk8 M.tb-infected mice 72 h post isotype control or anti-CD62L i.p. injection. (B) IFN-γ, TNF-α and IL-2 expression in CD4+ CD44+ T cells in the lung of control and anti-CD62L treated mice 72 h post isotype control or anti-CD62L i.p. injection. Data shown are representative of two independent experiments with similar results (n = 4 mice/group). Statistical differences between groups were determined by Student’s t test. *p < 0.05, ** p < 0.01. (TIF) [file ppat.1011460.s003.tif]
